# A catalogue of novel bovine long noncoding RNA across 18 tissues

**DOI:** 10.1371/journal.pone.0141225

**Published:** 2015-10-23

**Authors:** Lambros T. Koufariotis, Yi-Ping Phoebe Chen, Amanda Chamberlain, Christy Vander Jagt, Ben J. Hayes

**Affiliations:** 1 College of Science, Health and Engineering, La Trobe University Bundoora, Melbourne, Victoria, Australia; 2 Department of Environment and Primary Industries, AgriBio Bundoora, Melbourne, Victoria, Australia; 3 Dairy Futures Co-operative Research Centre, Melbourne, Victoria, Australia; Wageningen UR Livestock Research, NETHERLANDS

## Abstract

Long non-coding RNA (lncRNA) have been implicated in diverse biological roles including gene regulation and genomic imprinting. Identifying lncRNA in bovine across many differing tissue would contribute to the current repertoire of bovine lncRNA, and help further improve our understanding of the evolutionary importance and constraints of these transcripts. Additionally, it could aid in identifying sites in the genome outside of protein coding genes where mutations could contribute to variation in complex traits. This is particularly important in bovine as genomic predictions are increasingly used in genetic improvement for milk and meat production. Our aim was to identify and annotate novel long non coding RNA transcripts in the bovine genome captured from RNA Sequencing (RNA-Seq) data across 18 tissues, sampled in triplicate from a single cow. To address the main challenge in identifying lncRNA, namely distinguishing lncRNA transcripts from unannotated genes and protein coding genes, a lncRNA identification pipeline with a number of filtering steps was developed. A total of 9,778 transcripts passed the filtering pipeline. The bovine lncRNA catalogue includes *MALAT1* and *HOTAIR*, both of which have been well described in human and mouse genomes. We attempted to validate the lncRNA in libraries from three additional cows. 726 (87.47%) liver and 1,668 (55.27%) blood class 3 lncRNA were validated with stranded liver and blood libraries respectively. Additionally, this study identified a large number of novel unknown transcripts in the bovine genome with high protein coding potential, illustrating a clear need for better annotations of protein coding genes.

## Introduction

Analysis of transcripts within cells has revealed that up to 50% of the transcribed genome does not align to known protein coding regions, and many of these have no proven protein coding potential [1]. These non-protein coding transcripts can potentially be non-coding RNA (ncRNA), unknown RNA or transcriptional noise [2].

Non-coding RNA (ncRNA) are highly abundant and functional RNA molecules that are transcribed, but not translated into proteins. The ncRNA molecules found in the cell includes micro RNA, small inhibitory RNA and small nuclear/nucleolar RNA [3]. Recent advances in transcriptome sequencing has allowed for the discovery of a new class of ncRNA that are surprisingly long, known as long noncoding RNA (lncRNA) [4]. Long noncoding RNA are classified as having an arbitrarily defined length of more than 200 nucleotides (nts) with weak to no protein coding potential and largely have lower expression levels than messenger RNA (mRNA) [4, 5].

Long ncRNA share many characteristics with mRNA, they are transcribed by RNA Polymerase II, can be alternative spliced, are either single-exonic or multi-exonic, are differentially expressed and are usually (although not always) polyadenylated (PolyA(+)). These transcripts are thought to account for up to two thirds of the transcriptome in humans [4–6] and research is now focusing on understanding their functions, revealing that lncRNA have diverse roles in regulating epigenetic marks and gene expression [7]. Long ncRNA can also be post-transcriptionally processed to produce smaller RNA such as micro RNA [8], and increasing evidence suggests they are associated with enhancer regions [9]. These elements can be coded almost anywhere in the genome, within intergenic regions (also known as long intergenic ncRNA), within protein coding genes but on the opposite strand (known as antisense RNA) and within introns [10]. Pseudogenes are a recent addition to this list, believed to express long non-coding RNA that can have important regulatory roles on their protein coding counterparts [11].

Studies across a range of species, including humans [7], mouse [12], drosophila [13], *C*.*elegans* [14] and bovine [15, 16] are discovering many putative and potentially novel lncRNA across a growing range of tissues. Although one hypothesis is that some of these lncRNA could potentially be “transcriptional noise” or “transcriptional artifacts” due to RNA Polymerase II errors in elongation [17]. One of the best studied lncRNA examples is the *Xist* gene discovered across many species and functions to facilitate imprinting of the X chromosome [18]. Another well studied example is metastasis-associated lung adenocarcinoma transcript 1 (*MALAT1*) that is highly conserved in mammals. Functions range from regulating the expression of metastasis-associated genes [19] to transcriptionally regulating motility related gene expression (to promote cell motility) [20].

A common approach to discover and annotate putative lncRNA is to consider transcripts that have a nucleotide length greater than 200 nts, display moderate to high expression in tissues and show little to no evidence of protein coding potential [7, 8, 16, 21]. Criteria for the later varies greatly among studies, depending on the tool and methodology used. Each study typically defining their own thresholds for discriminating coding and noncoding transcripts. Unfortunately, due to the novelty of lncRNA, there is no concrete definition or methodology that allows for easy discovery of lncRNA. This ultimately leads to great variability in the number of putative lncRNA reported from different studies, even within the same species.

There are a number of studies reporting potential bovine lncRNA across many tissues using either EST data or RNA-Seq data [15, 16, 21, 22]. However, these catalogues are mostly limited to one or two tissues, and are not as comprehensive when compared to the repertoire of lncRNA found in human and mouse genomes. In this study we describe a comprehensive catalogue of putative bovine lncRNA expressed in 18 tissues and located within intergenic and pseudogene regions. Given the main challenge in identifying lncRNA is distinguishing them from transcripts of unannotated genes, we classified our putative lncRNA into 3 classes of increasingly stringent filtering, acknowledging that the more stringent filters may discard some true lncRNA. The class 3 transcripts were perhaps of the most interest since these passed stringent filters in the pipeline that show moderate to high expression levels. We compared our results to those from other bovine studies, along with lncRNA from mouse and human, to gain insights into the evolution of lncRNA across species.

## Results

Polyadenylated RNA from 18 tissues from one lactating cow were sequenced, three replicates per tissue with 40 to 100 million reads were generated per tissue, (S1 Table) [NCBI: SRP042639]. S1 Table also shows the number of generated reads that uniquely map to the UMD3.1 Ensemble reference genome. The tissues included; adrenal gland, black skin, white blood cells, brain caudal lobe, brain cerebellum, heart, kidney, leg muscle (semimembranosus), liver, lung, intestinal lymph node, mammary gland, ovary, spleen, thymus, thyroid, tongue and white skin.

Cufflinks [23] was used for annotation and transcript assembly with the bovine UMD3.1 Ensembl reference genome [24] and combined the output files using Cuffmerge into a single GTF file. Unknown transcripts were annotated using Cuffcompare, comparing the transcripts to the NCBI iGenomes reference gene library [25] to eliminate transcripts that have protein coding potential. We selected for transcripts that had either a class code of “u” (intergenic transcripts that have an unknown annotation) or “x” (transcripts that have exonic overlap with the reference genome but on the opposite strand). The Cufflinks/Cuffmerge/Cuffcompare pipeline resulted in a total of 47,117 transcripts with unknown annotations and nucleotide lengths ranging from 200 nts to 14,000 nts. Transcripts are defined as the locations on the reference genome that the assembled RNA-Seq reads align to.

**Fig 1:**
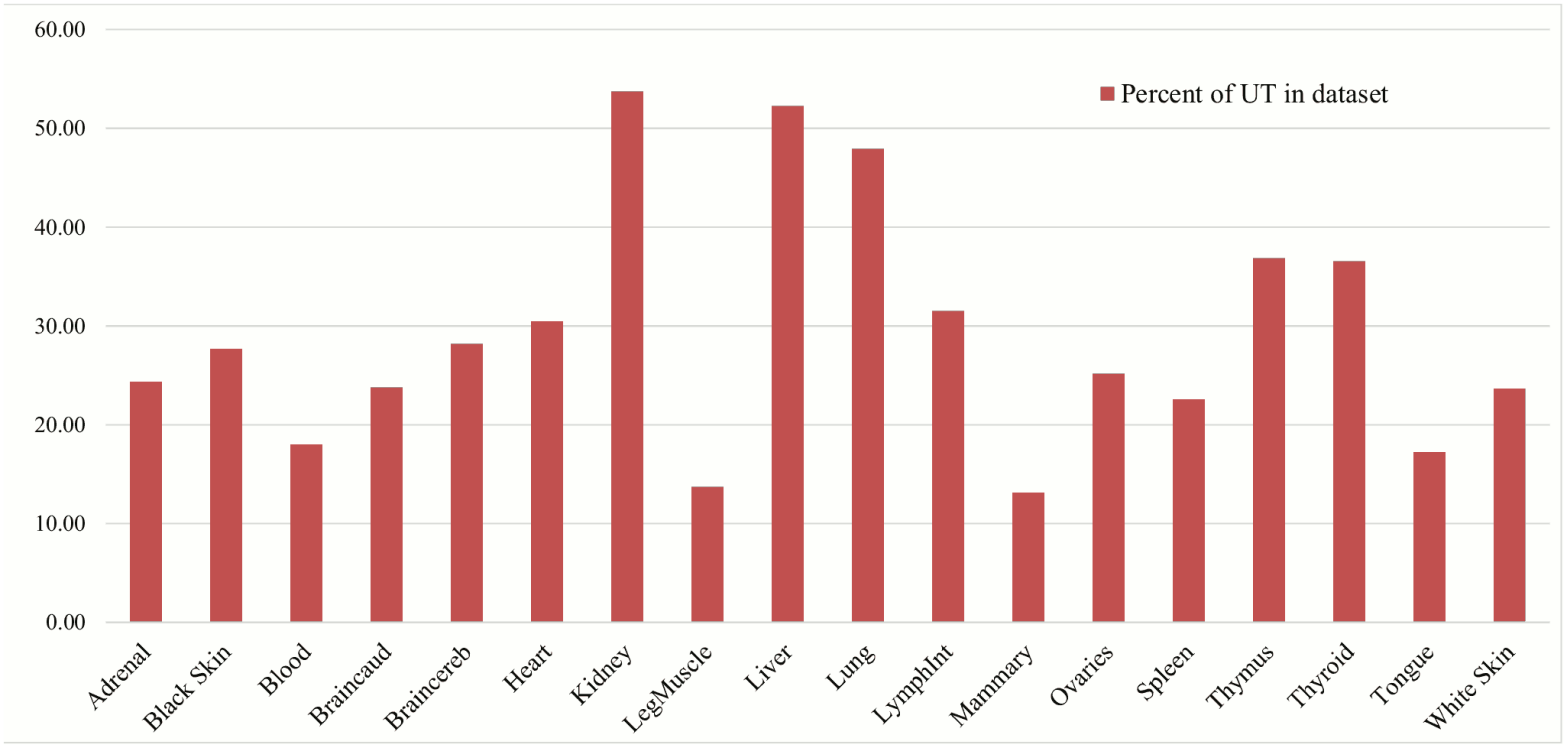
Percentage of intergenic assembled unknown transcripts (UT) out of total transcripts found for each tissue. This graph represents the percentage of unknown transcripts out of the total number of transcripts found after running the cufflinks pipeline on the RNA-Seq data. We see that the number of unknown transcripts discovered in our analysis fluctuates for each tissue, with kidney, liver and lung having some of the highest number of unknown transcripts. These could represent unknown novel RNA sequences, or artefacts and noise.

Fig 1 shows the percentage of transcripts that have an unknown annotation out of the total number of transcripts found across each tissue. The tissues kidney, liver and lung have some of the highest numbers of unknown transcripts in our dataset, while other tissues such as leg muscle (semimembranosus), mammary gland and tongue indicate that only 13.73%, 13.13% and 17.26%, respectively, of the transcripts in these tissues have unknown annotations. These unknown transcripts have the potential to be either novel RNA elements (either ncRNA or mRNA) or transcriptional artifacts.

The 47,117 unkown transcripts were passed through a filtering pipeline to find potential lncRNA transcripts. It is important to note that we have focused on novel lncRNA, so known and annotated bovine lncRNA are excluded from the final dataset. *Xist* and *MEG3* are examples of bovine lncRNA that are not found in our filtered data set since they are already annotated in the Ensembl bovine reference genome (both *Xist* and *MEG3* were in the unfiltered data set).

A detailed version of our pipeline workflow is described in the methods section (a workflow of the pipeline can be found in S1 Fig), and is broadly similar to pipelines used in similar studies [3, 7, 16, 21]. In brief the first step was to determine all possible open reading frame (ORF) in our unknown transcripts and then use the tool blastp to determine potential protein domains or similarities with other known protein sequences from the non-redundant protein sequence (nr) database. We found 20,927 unknown transcripts that had a nucleotide length of greater than 200 nts and do not show any sequence similarity with current known proteins from the nr database. The second filter in the pipeline used the tool blastx to find sequence similarities between the unknown transcripts with the nr database, resulting in a total of 16,584 transcripts that did not show sequence similarities. The final filter used the tools Coding Potential Calculator (CPC) [26] and CNCI [27] to determine coding or noncoding potential of the unknown transcripts. For stringent selection, we used a CPC coding potential score of < -0.5 as the threshold, the same value as used in a similar study [16]. From the CNCI results we selected transcripts with a predicted “noncoding” annotation. We also performed an alignment of the putative lncRNA with the pfam protein domain database [28], finding no protein domains.

Putative lncRNA transcripts were divided into three classes. Class 1 is for the transcripts that passed at least one of the three filters, finding a total of 24,381 transcripts. Class 2 represents putative lncRNA that passed at least 2 of the 3 filters in the pipeline, finding 20,301 transcripts. Class 3 were putative lncRNA that passed all 3 of the filters in the pipeline and had no protein domains when aligned to the pfam database (16,336 putative lncRNA). The R package EdgeR [29] was used to find and remove the transcripts that had a read count of less than 25 in any of the three replicate for each tissue. This final filtering step gave us a total of 9,778 class 3 putative lncRNA with moderate to high expression, Table 1. This should also be the class with the fewest false positives (transcripts that are not lncRNA). The correlation of expression of class 3 putative lncRNA across replicates for each tissue was very high (average correlation score of 0.98), with the exception of white skin which had a correlation score of 0.67 for one pair of replicates. Further, of the 9,778 class 3 lncRNA, 9,482 (96.98%) were annotated according to cufflinks as single-exonic, while 296 (3.03%) were multi-exonic. In the supplementary material, S2 Table shows the full list of potential class 3 lncRNA along with the number of exons for each transcript.

**Table 1:** Number of unknown transcripts that pass the filtering pipeline, showing no coding potential.

	Unknown transcripts with no coding potential	Filtered for moderate to high expression	Transcripts conserved with human lncRNA	Transcripts conserved with mouse lncRNA	Transcripts conserved with both human and mouse lncRNA
**Class 1**—Pass only one of the filters	24,381	9,828	396	115	53
**Class 2**—Pass at least 2 of the filters	20,301	10,467	645	186	57
**Class 3** –Pass all 3 filters	16,336	9,778	289	119	36

**Fig 2:**
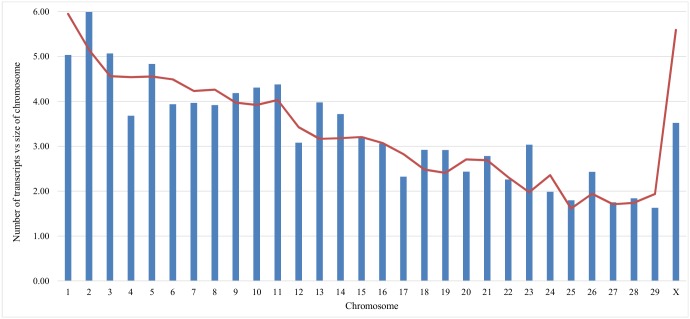
Total number of class 3 putative lncRNA per chromosome vs the size of each chromosome. This figure shows the correlation with the number of class 3 putative lncRNA found on each chromosome vs the actual size of our chromosome. The blue bars indicate the number of class 3 transcripts (as a percentage of the total number). The red line indicates the size of the chromosome (as a percentage of the total nucleotide size).

The genome distribution of putative lncRNA transcripts was investigated, Fig 2. Overall, there was a very high correlation between the number of class 3 transcripts and the size of the chromosome, correlation score 0.86. This indicates lncRNA are distributed across the chromosome in proportion to chromosome length.

### Expression patterns of lncRNA across 18 tissue samples in dairy cattle

**Fig 3:**
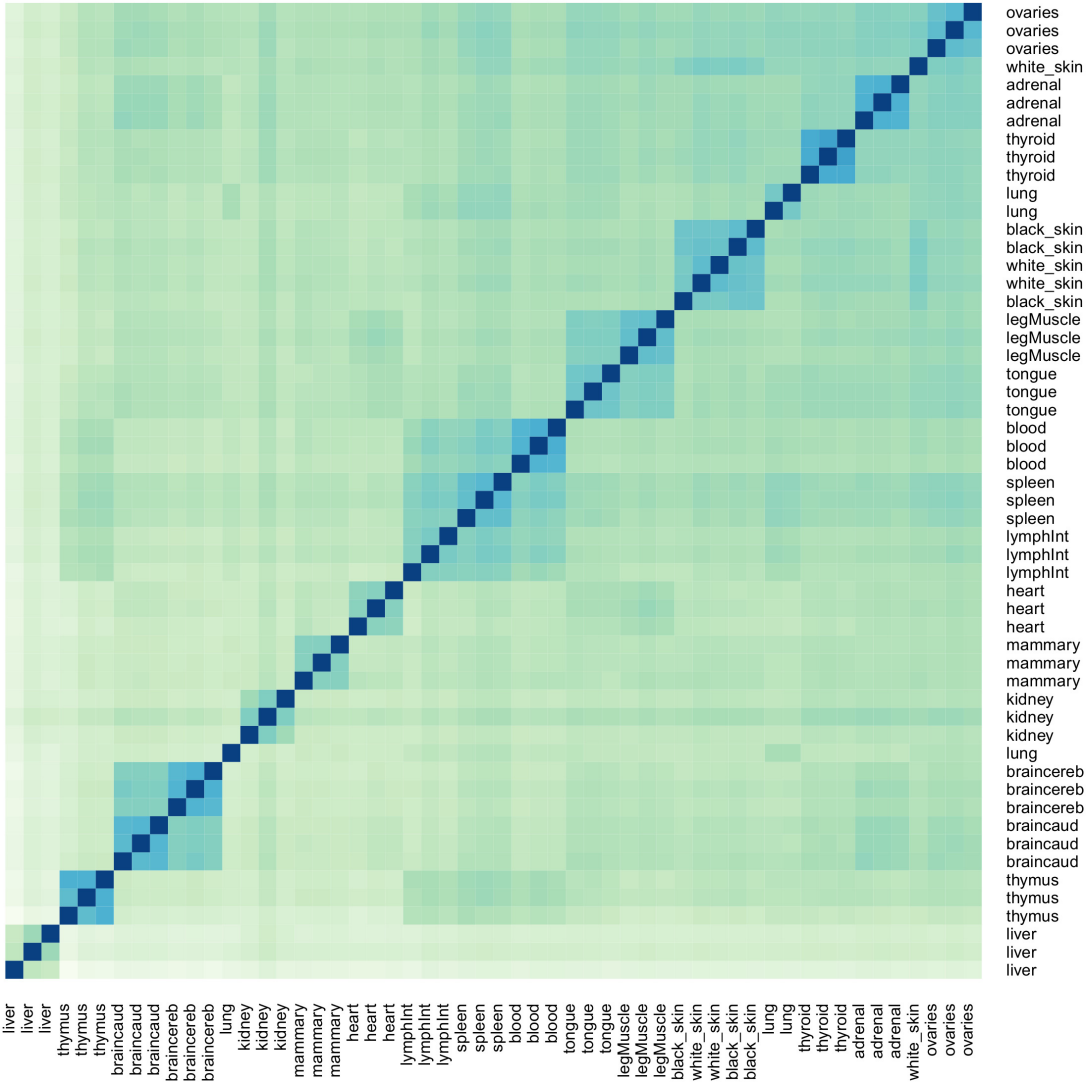
Tissue x tissue heat map and hierarchical clustering of gene co-expression data for putative intergenic long ncRNA. This heat map shows the number of transcripts that are co-expressed in each of the tissues in relation to another tissue along with the replicates (calculated using the package DESeq). The order of the tissues is based on their pairwise distances. The colour indicates the level of the expression correlation within tissue replicates and between tissue samples. The darker the blue colour is the higher the correlations are. A white colour indicates no similarities in the expression data.

To investigate expression patterns and hierarchical clustering of the class 3 putative lncRNA across tissues, the R package DESeq [30] was used, with input the counts data matrix generated from HTSeq and normalized using DESeq (Methods). The normalization was the DESeq standard, the scaling factor calculated as the median of the ratio, for each transcript, of its read count over the geometric read count for all samples [31]. We calculate the tissue x tissue along with the replicate x replicate gene co-expression (Euclidian distance), represented by a heat map in Fig 3. The tissues are ordered on the heat map based on their pairwise distances and we can clearly see that the tissues cluster into groups based on their biological function. The groups; Brain caudal lobe/brain cerebellum, white skin/black skin, intestinal lymph node/spleen/white blood cells, tongue/leg muscle cluster together due to having higher correlations (darker blue color) indicating these tissues are involved in similar organ functions. Our putative lncRNA cluster similarly to what was observed in protein coding transcripts across the 18 tissues from the same RNA-Seq dataset [32]. We also find that our putative lncRNA show significantly lower average and maximal expression levels across replicates and tissues than what was observed for the protein coding transcripts in that study [32], a property that lncRNA are known to possess [7].

**Fig 4:**
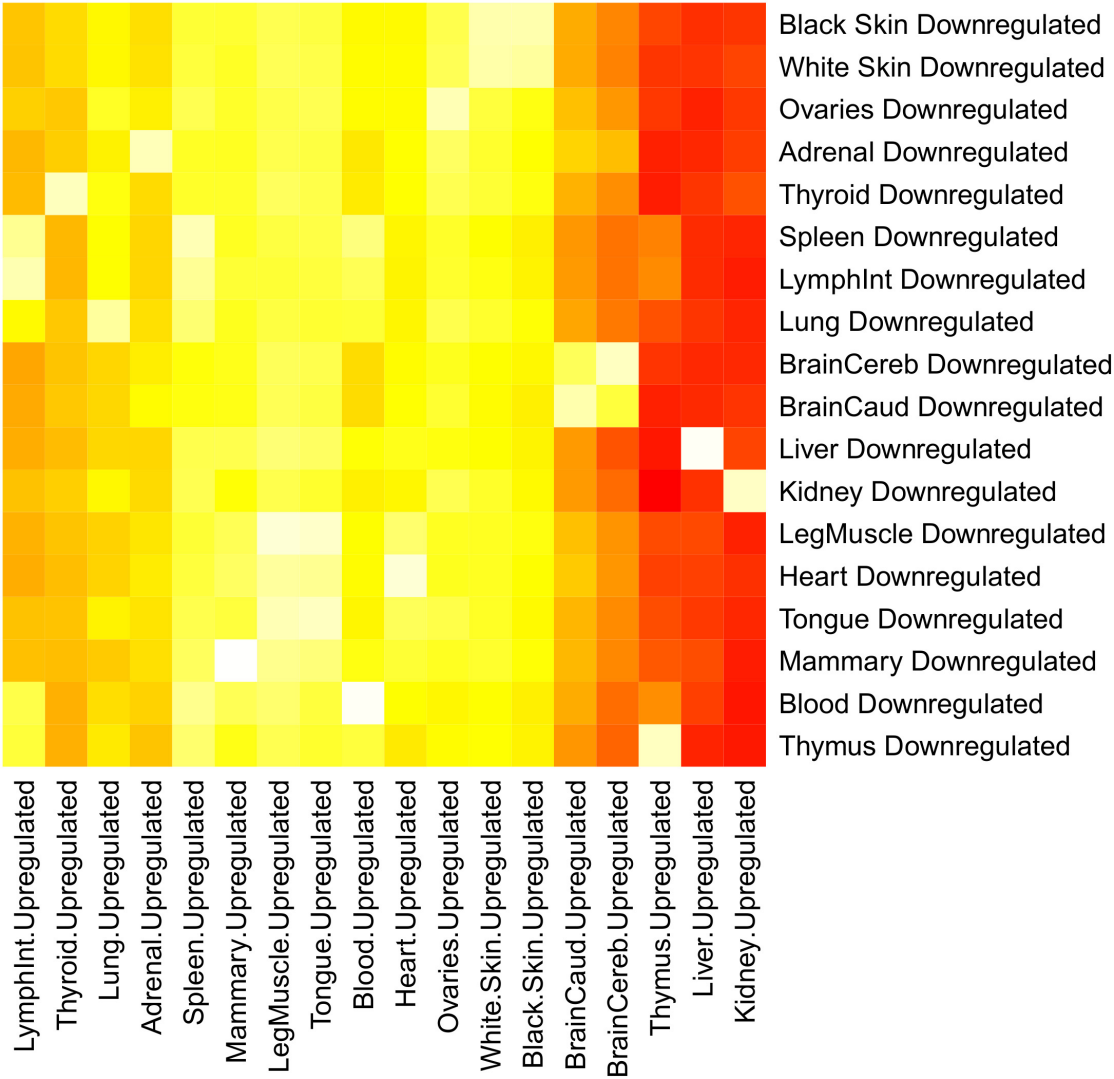
Differential expression heat map of class 3 lncRNA. This heat map shows the number of transcripts that either upregulated or downregulated for each tissue. On the x-axis are the upregulated tissues. On the y-axis are the downregulated tissues. The tissues are ordered and grouped based on upregulation. Red colors indicating the most differential expression, while white colors indicate the least differential expression.

The next step was to determine the number of class 3 lncRNA that are either upregulated, downregulated or show no differential expression in each tissue. To determine differential expression, we performed a pairwise differential expression analysis between two tissues using the package DESeq with the *nbinomTest* command. We determine a lncRNA as upregulated between two tissues if it had a negative log^2^ fold change and a *P-value* < = 0.05 (multiple testing corrected). If the log^2^ fold change was positive and the *P-value* < = 0.05 (multiple testing corrected) we considered that lncRNA to be downregulated in that tissue (Method). We noted the number of upregulated and downregulated transcripts between each tissue in a matrix shown in S3 Table, with the rows in the matrix defining the number of upregulated transcripts for that tissue, and the columns defining the number of downregulated transcripts for that tissue. Fig 4 shows the heat map of this matrix with the color red indicating a higher number of differentially expressed transcripts, and the color white indicating a lower number of differentially expressed transcripts. As we can see, kidney, liver, thymus, brain caudal lobe and brain cerebellum have a very large number of upregulated transcripts across almost all other tissues, indicating that upregulation in these tissues is overrepresented. Further, we see that the tissues tongue, white skin, spleen, ovaries and mammary show significantly less upregulation, indicating many transcripts that are downregulated in these tissues. Overall we see that on average the transcripts show levels of downregulation ranging from 131 to 267 while for upregulation ranging from 52 to 463 (S3 Table).

**Fig 5:**
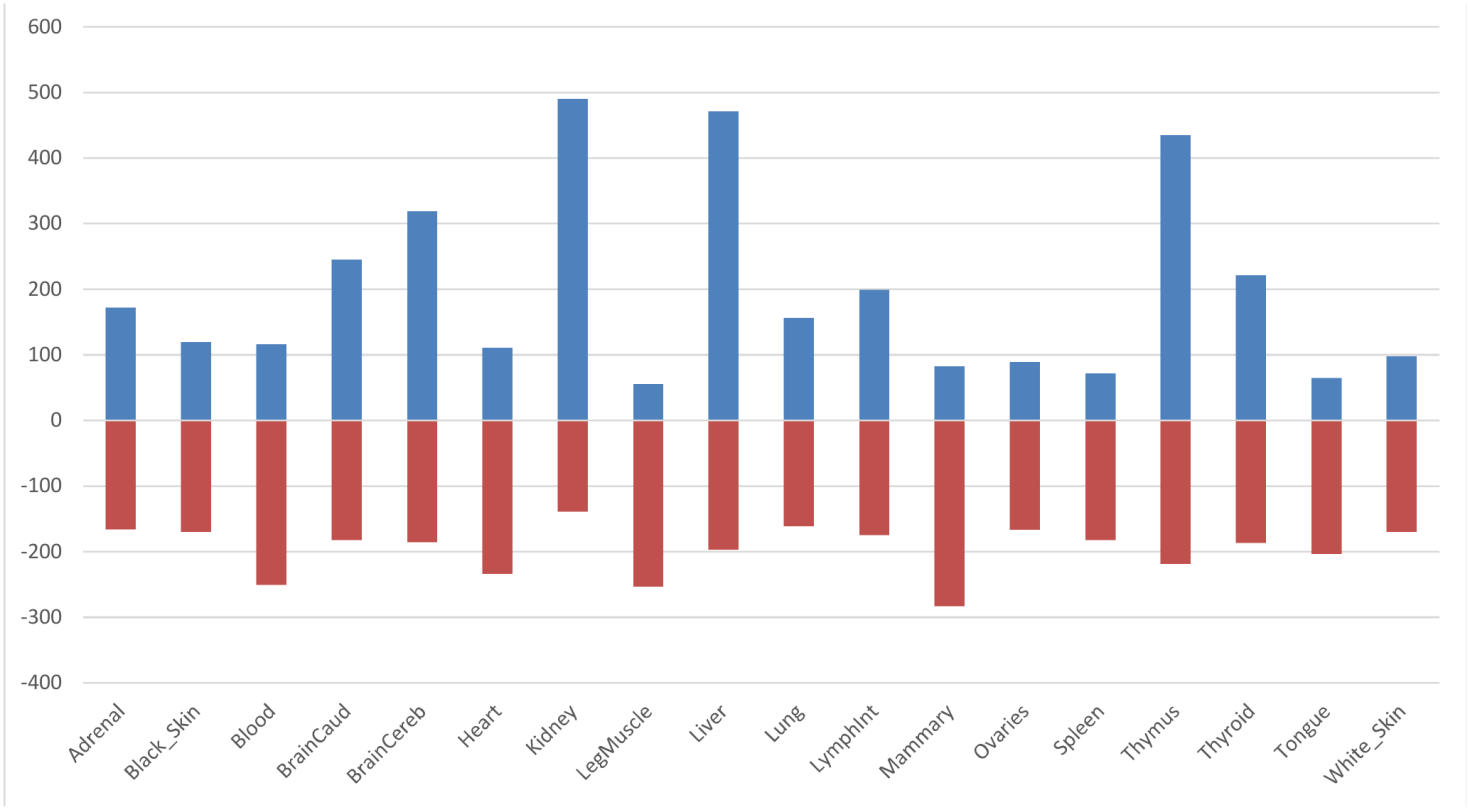
Average number of differential expressed class 3 transcripts that are either upregulated or downregulated. This graph shows us the average number of class 3 putative lncRNA transcripts that are either upregulated (blue bars) or downregulated (red bars). We see that in the tissues kidney, liver and thymus there are, on average, more upregulated transcripts, while in the tissues leg muscle, ovaries, spleen and tongue there is, on average, more downregulated transcripts.

Fig 5 reveals the average number of upregulated or downregulated transcripts for all tissues. The tissues kidney, liver and thymus have, on average, the highest number of lncRNA that are upregulated while blood, mammary and leg muscle show, on average, the most downregulated lncRNA. Combined with what we saw in the results from Fig 4, this reveals that these tissues, particular liver, kidney, thymus and mammary have some of the most differentially expressed transcripts in our analysis.

### Comparative analysis with human and mouse lncRNA

The class 3 putative lncRNA were compared to human and mouse ncRNA databases to gain insights into homology. Human and mouse lncRNA sequences from GENCODE version 7 [33], NONCODE v4 [34] and from lncRNAdb [35] were obtain and the tool blastn was used with an E-value of 1x10^-6^ as the threshold to determine sequence similarities.

Of the 47,117 unknown bovine transcripts, a total of 4,831 showed significant sequence similarity with at least one lncRNA in either the human or mouse databases. Of the 9,778 class 3 putative lncRNA transcripts, 289 showed significant sequence similarity with known human lncRNA and 119 showed significant sequence similarity with known mouse lncRNA (full list can be found in S4 Table). Further, only a mere 36 putative lncRNA showed sequence similarity with known lncRNA in both human and mouse (Table 1). It has been suggested that lncRNA evolve at a more rapid rate than mRNA with studies finding only 12% of human and mouse lncRNA are conserved in other species [36], a possible explanation for why very few bovine lncRNA transcripts are conserved with human and mouse lncRNA.

Curiously some of the transcripts that have protein coding potential, showed significant sequence similarities with human or mouse lncRNA (of which we find 15,590). One example of this observation is the transcript *XLOC_048061* found upstream of the transmembrane protein 245 gene on chromosome 8. Significant sequence similarity was found with two human lncRNA from the NONCODE database; *NONHSAT133928* (E-Value 0.0) and *NONHSAT133929* (E-Value 2x10^-77^) both of these lncRNA are found on chromosome 9 in the transmembrane protein 245 coding region. However, this transcript also showed very strong sequence similarity with the transmembrane protein 245 in many mammalian species including; *Bos taurus*, *Bubalus bubalis*, *Ovis aries and Physeter catodon*. These findings could indicate the possibility that it can either be an un-annotated alternative exon transcript for the transmembrane protein 24 or a potential lncRNA. However, further analysis would be needed to reliably annotate it.

### Specific Examples

#### MALAT1 region

MALAT1 is a lncRNA involved in regulation of cell motility and cancer metastasis. We discovered a potential MALAT1 region in our dataset on chromosome 29 at loci 44,337,428–44,345,287 with ID XLOC_034725, having a nucleotide length of 7,859 a CPC score of -0.179 and a CNCI prediction of “noncoding”. However, when the blastx tool was used, it predicted a MALAT1 protein domain, placing this transcript in class 2 instead of the final class 3. We used the tool blastn to compare this transcript with the nucleotide collection database finding sequence similarities with a misc_RNA annotated transcript, NCBI reference sequence XR_240684.1. Significant sequence similarities was also found with the human MALAT1 region showing a blast e-value of 0.0 and an identity of 83% and with the mouse MALAT1 region showing an e-value of 0.0 and an identity of 80%. This region is not annotated as MALAT1 in the Ensembl bovine UMD3.1 reference genome. A previous study using RNA-Seq data, found a number of reads mapped to a very similar region in the bovine genome that was determined to potentially be the MALAT1 region [15].

#### HOTAIR region

Another lncRNA of great interest is *HOTAIR*, a lncRNA that has been described in humans, located near the HOXC gene cluster and abundantly expressed in cancer cells [37]. A class 3 transcript (ID: *XLOC_040767)* on chromosome 5 at the locus *26*,*224*,*347–26*,*229*,*423* was found to be a potential *HOTAIR* candidate. This transcript had a nucleotide length of 5076 nts, a CPC score of -0.785, a CNCI prediction of “noncoding” and no presence of a substantial ORF that has any significant sequence similarity with protein sequences. After using the tool blastn to compare the transcript with the nucleotide collection database, significant sequence similarity was found with both human (e-value 4x10^-129^, 75% identity) and mouse (e-value 1x10^-07^, 100% identity) *HOTAIR* lncRNA sequences. As with *MALAT1* this region is not annotated in Ensembl UMD3.1 reference genome. Sequence similarity was also found with a bovine miscellaneous RNA labelled *LOC101907241* (NCBI reference of *XR_234518*.*1*).

#### XLOC_035708 region

This locus is of particular interest because the pipeline reveals no protein coding potential, yet in NCBI it is predicted as an mRNA for the potassium channel, voltage-gated, shaker-related subfamily A, member 3 (*KCNA3*) gene. Found on chromosome 3, it is located downstream of the *KCNA3* gene by more than 14,000 nucleotides, with a nucleotide length of 5,915 nts, a low CPC score of -1.187, a “noncoding” CNCI prediction and an ORF that did show significant sequence similarity with known protein sequences. We found significant sequence similarity (e-value = 0.0, identity = 78%) with a human lncRNA (*ENSG00000259834*) that is a *KCN3* lncRNA found on chromosome 1, and also significant similarity (e-value 1x10^-142^, identity 79%) with a mouse lncRNA (*ENSMUSG00000056145*) that is found downstream of the *KCN3* gene on chromosome 3. We predict that this locus on the bovine genome could potentially be a lncRNA conserved in both the human and mouse genomes and can have a regulatory role for the *KCN3* protein. The expression of this locus is quite high with an average normalized expression count of 815.84 (across all tissues), while the *KCNA3* protein coding gene has a significantly lower normalized expression level of 39.82 (across all tissues).

#### XLOC_010252 region

This transcript found on chromosome 13 at the locus *41*,*890*,*255–41*,*891*,*513* with a length of 1,258 nts is of great interest because it is located downstream of the forkhead box A2 (*FOXA2*) gene in the bovine genome. *FOXA2* is a transcription factor (TF) that regulates the expression of genes involved in glucose sensing [38]. We found this putative lncRNA had significant sequence similarity with the human lncRNA *ENSG00000259974* (e-value 0.0 and identity 91%), and the mouse lncRNA *ENSMUSG00000086141* (e-value 2x10^-180^ and identity 88%). Both the human and mouse lncRNA are located downstream of the forkhead box A2 (*FOXA2*) transcription factor binding site (TFBS) and are suggested to have a role in gastric cancer [39]. There is evidence suggesting that lncRNA are preferentially found near (either upstream or downstream) gene deserts surrounding transcription factor genes. The lncRNA flanking transcription factor genes can have important *cis* regulatory roles in gene expression and lncRNA that are found near TF genes can act together in co-regulation [36]. Also, a lncRNA can recruit transcription factors to activate certain genes [1]. We predict, that this transcript can potentially be a lncRNA involved in regulating the genes involved in glucose sensing by interacting with the *FOXA2* transcription factor.

### Long non coding RNA found near coding genes

While the majority of the class 3 lncRNA are found in intergenic islands, some are found near known protein coding genes. A total of 1,547 (15.82%) class 3 putative lncRNA were located either 5 kilobases (kb) upstream or downstream of genes or slightly overlapped a protein coding gene at the 3 prime or 5 prime end (full list can be found in S5 Table). These transcripts have the potential be lncRNA, particularly antisense RNA (asRNA) if found on the opposite strand of the protein coding gene. They can also represents un-annotated exons from an alternative transcript of the protein coding gene.

One method to predict lncRNA close to genes is to measure the concordance of expression between the lncRNA transcript and the neighboring protein coding genes. This would indicate that the higher the correlation is between two transcripts, the more likely they are to be from the same gene. To perform this analysis we calculated the Pearson product-moment correlation coefficient (Pearson’s correlation) to determine the linear measure between the class 3 lncRNA expression and its neighboring protein coding gene expression. We also calculated the Spearman’s rank correlation coefficient (Spearman’s rho) to measure the similarity in the rank of expression.

**Fig 6:**
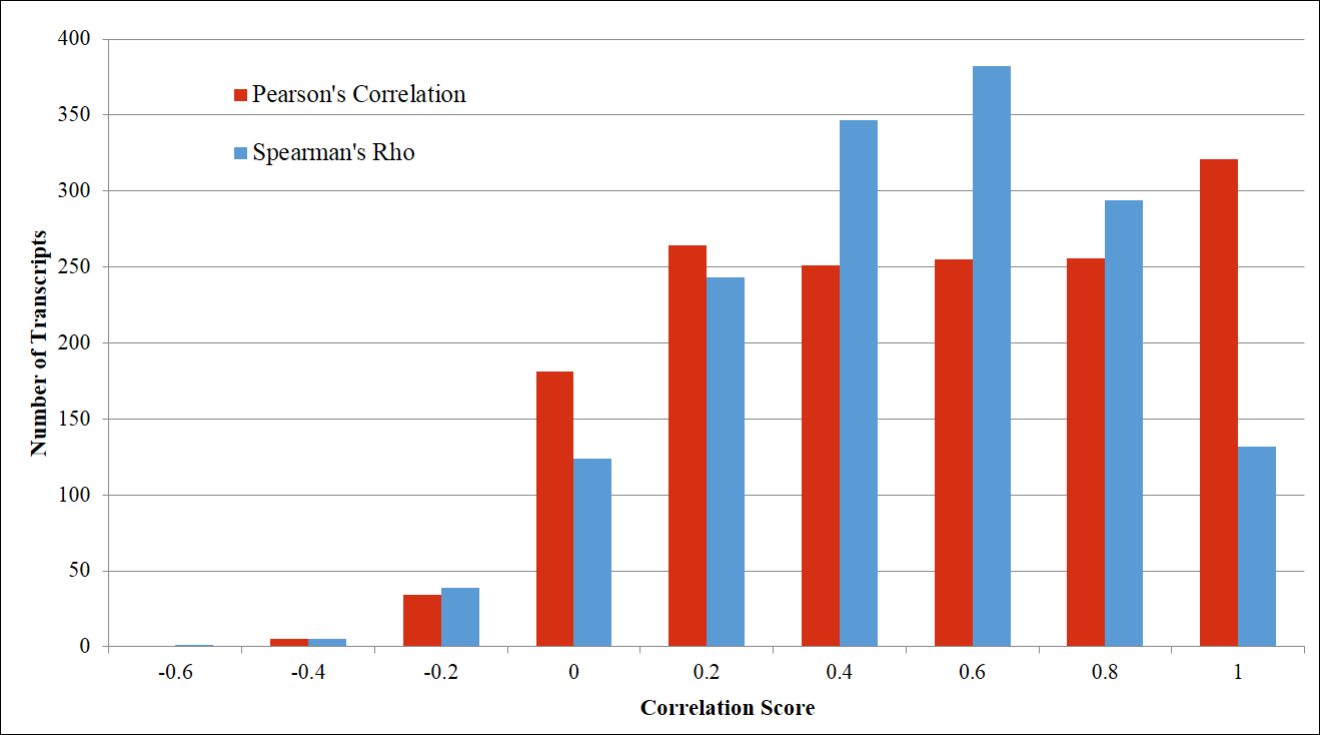
Correlation analysis between the expression patterns of the putative lncRNA transcripts and the neighboring protein coding transcripts. The Pearson’s correlation anlysis is represented by the blue bars. Spearman’s rho is represented by the orange bars. We considered a cut off for the level of correlation to be <0.6 for a lncRNA/mRNA pair that is uncorrelated

For the Pearson’s correlation analysis we observe (blue bars, Fig 6) many class 3 lncRNA have high correlations with scores of 0.60 and above. A similar trend can be seen in the Spearman’s rho (orange bars) with even more lncRNA transcripts having high correlation scores of 0.60 and above. The lncRNA with high correlations can potentially be either RNA artifacts from the protein coding gene or un-annotated protein coding transcripts. Looking at the number of lncRNA that have low correlations (a score of less than 0.60) we see that in both the Pearson’s correlation and Spearman’s rho many lncRNA do not share a similar expression as that of the neighboring protein coding gene. These would be of most interest for further analysis, since the correlations scores indicate independent coding potential.

### Validation of lncRNA in with blood and liver stranded RNA-Seq, including in additional animals

Almost all tissue samples in this study are limited by the fact that we do not have stranded information to determine the coding direction of the transcripts. However, stranded RNA-Seq data was available for the tissues blood and liver. For liver, the stranded RNA-Seq libraries came from the same cow that we obtained the 18 tissue samples from, while for blood the stranded RNA-Seq libraries came from 3 additional cows. We used the stranded information for validation of the class 3 lncRNA, particularly for those found near protein coding genes.

The transcripts from the stranded liver RNA-Seq reads were obtained using both TopHat and fastQC to align the reads to the bovine genome and for quality control, while a Cufflinks/ Cuffcompare pipeline was used for transcript reconstruction and annotation (Methods). We then determined the number of transcripts from the stranded liver libraries that overlapped with the class 3 lncRNA. In total we found 830 liver transcripts with stranded information overlapping the 9,778 class 3 lncRNA and 726 (87.47%) liver class3 lncRNA overlapped with the validated stranded liver transcripts. Further, of the 1,547 class 3 lncRNA found near protein coding genes, 115 (7.43% of the 1,547) were validated with the liver stranded libraries. A further 50 (43.78% of the 115) of the validated lncRNA had a coding direction in the opposite strand of the neighboring protein coding genes.

#### Validation using stranded RNA-Seq reads from blood in 3 additional cow

The blood stranded RNA-Seq reads were available from 3 additional cows with ID’s 210004817, Y10ST0027 and Y10ST0106. As what was done with the liver stranded RNA-Seq reads, TopHat and fastQC was used for quality control and to align the reads to the bovine genome and a Cufflinks/Cuffcompare pipeline was used for transcript reconstruction and annotation (Methods). Next was to determine the number of stranded blood transcripts that overlap with the 3,018 class 3 blood expressed lncRNA. Table 2 shows us the number of validated lncRNA from the 3 additional cows that overlap with the un-stranded class 3 lncRNA. 26.34% of the validated lncRNA from the cow 210004817 overlap specifically with the blood class 3 lncRNA. 16.57% of the validated lncRNA from the cow Y10ST0027 overlap specifically with blood class 3 lncRNA. Finally, 43.97% of the validated blood lncRNA from the cow Y10ST0106 overlap specifically with the blood class 3 lncRNA. Overall we find a total of 2,508 validated stranded blood transcript that overlap with the class 3 lncRNA and of these 1,668 (55.27%) overlap specifically with the blood class 3 lncRNA (Table 2).

**Table 2:** Validated lncRNA from blood stranded RNA-Seq that overlap with class 3 un-stranded lncRNA.

Cow ID	Overlap with class 3 lncRNA	Overlap only with blood class 3 lncRNA (3,018)	Overlap with class 3 lncRNA close to protein coding genes (1,547)	Have coding direction on opposite strands to protein coding genes
210004817	1,076	795	158	92
Y10ST0027	630	500	90	40
Y10ST0106	2,057	1,327	293	153
Combined	2,508	1,668	390	198

Next, was to examine the proportion of the validated class 3 blood lncRNA that are found close to protein coding genes, since these have the potential to be antisense RNA. For the 210004817 cow, a total of 158 (10.21% of 1,547) of the blood validated class 3 lncRNA were found to be close to protein coding genes, with 92 (58.23% of 158) having a coding direction opposite to that of neighboring protein coding genes (Table 2). For the Y10ST0027 cow, a total of 90 (5.82%) blood validated class 3 lncRNA were found close to protein coding genes and only 40 (44.44%) having an opposite coding direction to a protein coding gene. Finally, for the cow Y10ST0106 we find 293 (18.94%) blood validated class 3 lncRNA close to protein coding genes with 153 (52.22%) of these having a coding direction opposite to the protein coding gene. The combined number of blood validated class3 lncRNA that are found near protein coding genes is 390 (25.02%) with 198 having coding directions opposite to protein coding genes (Table 2). The full list of blood validated class 3 lncRNA that are found near protein coding genes can be found in S6 Table.

**Fig 7:**
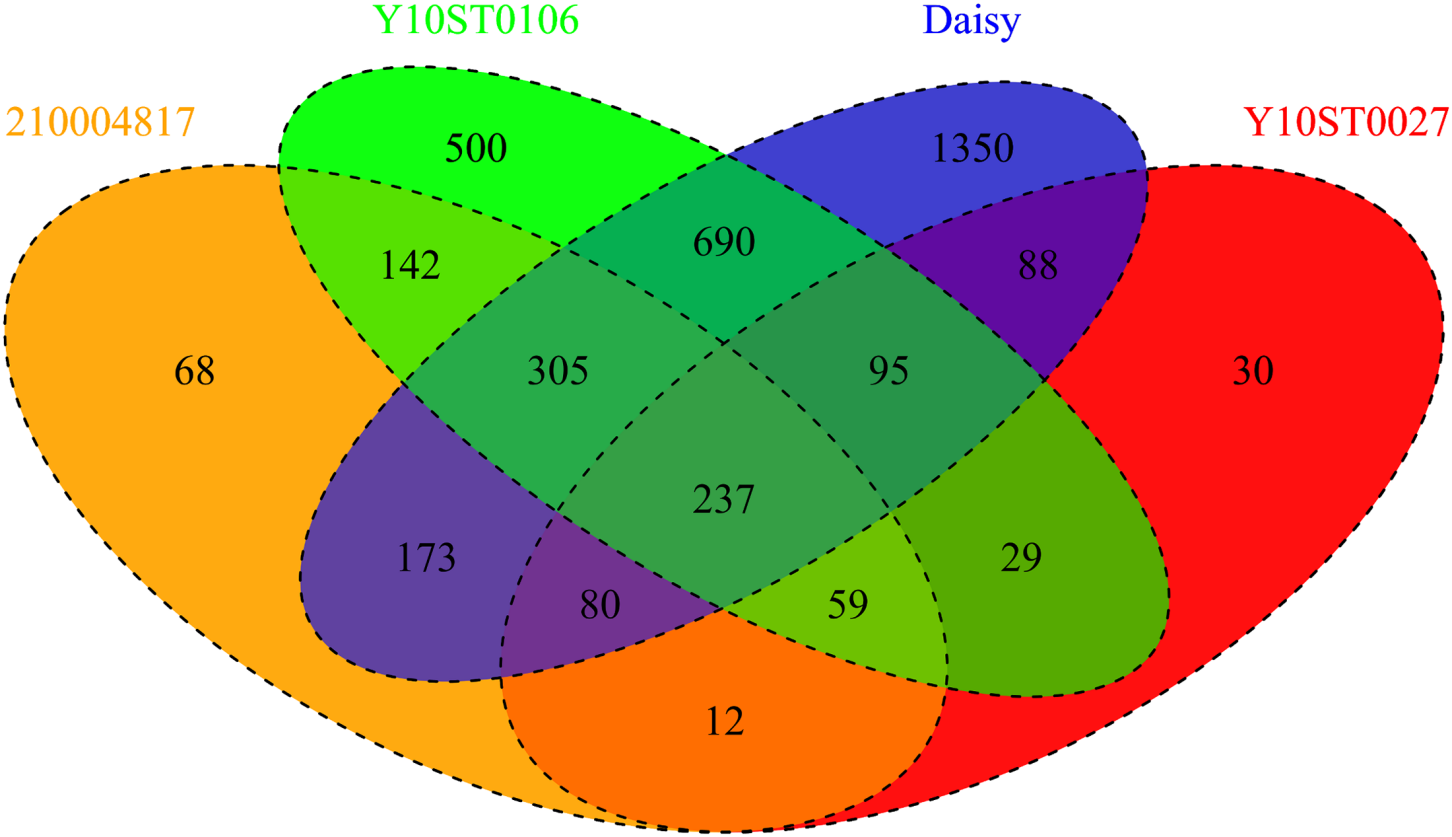
Venn diagram of number of validated class3 lncRNA with stranded blood libraries that are found between each animal. This figure represents the number of common and unique class 3 lncRNA validated with stranded RNA-Seq from blood that are found in each animal. The green circle represents the validated lncRNA found in the cow 210004817. The orange circle represents the validated lncRNA found in the cow Y10ST0106. The blue circle represents the class 3 lncRNA found in the cow daisy. The red circle represents the validated lncRNA found in the cow Y10ST0027. In the very middle we see that 237 validated class 3 lncRNA are found in all 4 animals.

Fig 7 shows a Venn diagram of the overlap of the validated blood class 3 lncRNA that is found between each animal, including the blood lncRNA from daisy (the cow where our 18 tissue samples came from). Of the 2,508 blood lncRNA, about 1,350 are specific to the cow daisy. In the cow Y10ST0106, 500 blood lncRNA are found exclusively only to that animal, while the other two have significantly lower unique transcripts (68 in cow 210004817 and 30 in cow Y10ST0027). The total number of validated blood lncRNA that are found in all 4 cows is 237, which is 15.32% of the total class 3 lncRNA found near protein coding genes and 2.42% of the total class 3 lncRNA. It is quite likely that the lncRNA that do not overlap reflect some cow to cow variation in lncRNA expression. This analysis would need further work to determine if these transcript have the potential to be antisense RNA and we anticipate to expand on this work in future research.

### Pseudogenes coding for lncRNA

Pseudogenes are found in many mammalian genomes that resemble functional genes, but due to the result of accumulating mutations, have lost their functions. While these are “inactive” genes, there is increasing evidence that lncRNA might arise or evolve from pseudogenes [36]. Here, we attempted to find bovine pseudogenes that can potentially have lncRNA functions. To our knowledge lncRNA studies in bovine genomics have not considered this. To do this we assembled RNA-Seq reads that aligned to known pseudogenes from Ensembl version 75 using Cufflinks and then used HTSeq to obtain the read count of the assembled transcripts that aligned in or near pseudogenes. A total of 798 pseudogenes in the bovine genome had RNA-Seq reads mapping to them, however it is important to note that some of the RNA-Seq reads from the parental genes or paralogues of the pseudogenes could potentially incorrectly map to the pseudogene loci. Therefore this leads to mapping ambiguity in the expression data, since some pseudogenes might appear to be more expressed than they really are. However, in this analysis we are not attempting to quantify the expression of pseudogenes but instead to merely identify the pseudogenes that are more likely to harbor lncRNA by using the expression data as a filter.

EdgeR was used to remove assembled pseudogene transcripts that had low read counts and select for those that had moderate to high expression across each replicate for all 18 tissues. This reduced the number of transcripts to 329 pseudogenes with moderate to high expression patterns (full list is found in S7 Table). As was mentioned above, due to mapping ambiguity some of these pseudogenes might be incorrectly showing expression. Therefore we perform a conservation analysis to filter out only the transcripts that have significant sequence similarities with either a human or mouse lncRNA. After applying the conservation analysis a further 255 pseudogenes were found to potentially have lncRNA.

One of the best examples in our study of a pseudogene that has the potential to code for a lncRNA is *ENSTBTAG00000009359*, a pseudogene for the ferritin heavy chain gene found on chromosome 15 at the locus *38*,*876*,*122 to 38*,*876*,*667*. Our analysis found that this region had reads that aligned outside of this locus, beginning at *38*,*869*,*503* to *38*,*876*,*667*, showing significant sequence similarities with the human ferritin heavy polypeptide 1 pseudogene 3 (*FTH1P3*) ncRNA (e-value 9x10^-97^ and identity 90% with NCBI reference sequence *NR_002201*.*1*) and with the mouse ferritin heavy chain 1 (*Fth1*) transcript variant 2, lncRNA (e-value 0.0, identity 86% with NCBI reference sequence *NR_073181*.*1*). This indicates that this pseudogene might have a lncRNA that is conserved in both human and mouse.

Table 3 shows a list of the most highly expressed transcripts, some of which had reads that mapped outside of the Ensembl defined locus for that pseudogene and also had human or mouse lncRNA sequence similarities, the full list can be found in S7 Table.

**Table 3:** Pseudogenes with transcripts that are moderately to highly expressed, and show significant sequence similarity with a lncRNA.

Pseudogene	Ensembl defined loci	Cufflinks predicted loci	Conserved Protein from Blastx	Conserved lncRNA
*ENSTBTAG00000009359*	*chr15*:*38*,*876*,*122–38*,*876*,*667*	*Chr15*:*38*,*869*,*503–38*,*876*,*667*	Ferritin heavy chain gene	*NR_002201*.*1* (human)
				*NR_073181*.*1* (mouse)
*ENSBTAG00000046307*	*chr14*:*20*,*738*,*814–20*,*740*,*407*	*chr14*:*20*,*738*,*690–20*,*741*,*057*	enhancer-binding protein	*ENST00000587312*
*ENSTBTAG00000000609*	*chr1*:*54*,*654*,*216–54*,*654*,*795*	*chr1*:*54*,*649*,*544–54*,*654*,*834*	60S ribosomal protein L10, partial	*NONHSAT091397*
				*NONMMUT040103*
*ENSTBTAG00000030281*	*chr11*:*104*,*186*,*563–104*,*187*,*141*	*chr11*:*104*,*185*,*811–104*,*188*,*790*	ribosomal protein L9-like	*NONBTAT002126*
				*NONSAT064119*
				*NONMMUT029554*
*ENSTBTAG000000337913*	*chr28*:*44*,*925*,*709–44*,*927*,*696*	*chr28*:*44*,*921*,*657–44*,*930*,*341*	zinc finger protein 22	*NONHSAT013060*
*ENSTBTAG00000007807*	*chr10*:*76*,*992*,*202–76*,*994*,*112*	*chr10*:*76*,*992*,*081–76*,*996*,*921*	heat shock-related 70 kda protein	*NONBTAT002672*
				*ENSMUST00000151136*
				*NONMMUT029382*/3
*ENSTBTAG00000021135*	*chr8*:*78*,*599*,*030–78*,*600*,*903*	*chr8*:*78*,*586*,*061–78*,*604*,*284*	recQ-mediated genome instability protein 1	*NONBTAT024698*
				*NR_031761*.*1* (mouse)
*ENSTBTAG00000013311*	*chrX*:*94*,*378*,*601–94*,*380*,*496*	*chrX*:*94*,*378*,*159–94*,*380*,*748*	eukaryotic peptide chain release factor GTP-Binding subunit	*NONBTAT026758*
				*NONHSAT023985*
				*ENSMUST00000181526*
				*NONMMUT000613*
*ENSBTAG00000016116*	*chr12*: *33*,*647*,*815–33*,*648*,*405*	*Chr12*: *33*,*647*,*815–33*,*648*,*405*	MARCKS-related protein 1 (MARCKSL1)	*NR_052852*.*1* (human)
				*NR_028405*.*1* (mouse)

### Comparative analysis with bovine lncRNA from similar studies

A comparative analysis of our dataset with the putative lncRNA found in similar studies was performed to determine overlap. We obtained the lncRNA from the studies by C. Billerey *et al*. [21], R. Weikard *et al*. [16], Z. Qu & D.L.Adelson [15] and also examined lncRNA found in database: A domestic-animal long noncoding RNA database (ALDB) [40]. In house python scripts were developed to determine the number of class 3 transcripts that overlap with the bovine lncRNA from these studies by looking at the sequence locations in the reference assembly.

In the paper by Weikard *et al*. [16] used bovine skin pigmented and non-pigmented tissues to find lncRNA. A total of 848 (out of 4,948) transcripts were found to overlap with our class 3 transcripts. In the paper [21] that used bovine muscle tissue to find lncRNA, we find a total of 129 (out of 584) transcripts overlap with our class 3 putative lncRNA, interestingly 567 transcripts from the unknown transcript dataset map to the 584 lncRNA form that study, which is a relatively high proportion.

The paper [15] used expression sequence tags (EST) to find lncRNA across the whole genome, and has some of the most systematic identified lncRNA in bovine genomics. Since that study used the bosTau4 assembly (we used the bosTau6), we obtained the fastA sequences from the 23,060 predicted lncRNA of that study by using the tool twoBitTofa and align them to the fastA sequences from the class 3 lncRNA with the tool BLAT [41]. The transcripts that displayed very high sequence similarities (had a minimum sequence identity of >95) were only considered. We find a total of 287 class 3 lncRNA that overlap with the 23,060 predicted lncRNA from that paper. A very small number.

The ALDB [40] is a comprehensive database of lncRNA found across different breeds of domestic animals, including bovine. Currently, a total of 8,250 bovine lncRNA are annotated in this database. We found that 1,254 of the class 3 lncRNA overlap with the bovine lncRNA from that database. Interestingly enough, 1,179 of the 1,254 class 3 lncRNA that do overlap, were single-exonic while a mere 75 were multi-exonic, indicating that single-exonic lncRNA have the potential to be more conserved and prevalent in the bovine genome.

## Discussion

In this paper, we have produced a comprehensive map of bovine lncRNA, using polyA(+) captured RNA-Seq data across 18 tissues from a single lactating cow. Our results suggest pervasive tissue specific expression of lncRNA. We also validated the lncRNA expressed in some tissues using stranded RNA-Seq information in the tissues blood and liver to gain further insights into how these lncRNA are expressed, particularly when we consider their proximity to protein coding genes. To our knowledge, this is the first bovine lncRNA study to include such a diverse number of tissue types, finding 9,778 class 3 putative lncRNA and a further 255 bovine pseudogenes with potential to have lncRNA.

Due to the strict selection of the filtering pipeline, there remains a large number of transcripts not present in the final class 3 dataset, found in class 2 and class 1, which have the potential to be true lncRNA. However, many of the transcripts in class 1 and class 2 could be un-annotated protein coding RNA or just random RNA transcriptional errors from RNA Polymerase II (known as transcriptional artifacts). Ultimately, this is the cause for one of the greatest weaknesses in our study, since in the bovine genome many unannotated genes exist, both protein and non-protein coding. This makes it very difficult to provide further annotations to most of the unknown transcripts in our data. Experimental strategies along with further experimental data will be needed to provide further evidence of transcript initiation and elongation along with determining exon-intron structures [36]. To help minimize the number of transcriptional artifacts, a combination of cap analysis of gene expression (CAGE) could further assist in determining transcription start sites while 3P-seq data would assist in finding polyadenylation sites for determining the end of a transcript [36]. Projects such as the Functional Annotation of Animal Genomes (FAANG) that aim to provide a map of functional elements in the genome of domesticated animals, will also further help to provide reliable and extensive annotations of genes in the bovine genome, and would greatly help to further discriminate potential bovine lncRNA [42].

LncRNA are known to be either single-exonic or multi-exonic and are also known to possess fewer exons and display less efficient splicing than protein coding genes [7]. It has also been suggested that single-exon lncRNA are more likely to be conserved when compared to human-specific datasets [43]. However, a challenge in discovering lncRNA is to distinguish them from the abundant lowly expressed, single-exonic fragments from RNA-Seq data [7]. One effective approach to reduce this number of lowly expressed RNA-fragments is to filter out the transcripts that are single exonic keeping only multi-exonic transcripts [36]. Nonetheless, applying such a filter could also potentially discard many true lncRNA that are single-exonic, such as *MALAT1* and *NEAT* [36]. A study that examined lncRNA in both human and mouse find a large number of the lncRNA transcripts are single-exonic [34]. In another similar study that examine lncRNA in bovine skin tissue, a large majority of the predicted lncRNA are single-exonic [16]. We acknowledge that filtering transcripts based on exon numbers has been suggested to remove RNA fragments that are artefacts, however applying such a filter could potentially remove many lncRNA. In this study we chose to include both single-exonic and multi-exonic lncRNA for our analysis, as was done in other studies, and depended on the filtering pipeline and quality control to reduce the number of RNA artefacts.

Discovery of lncRNA is complicated further with evidence that some lncRNA having the ability to code for polypeptides. One such example is found in Zebrafish where the *steroid receptor RNA activator 1 (sra1)* gene, initially thought to be non-coding, not only codes for a ncRNA but has been found to code for the protein steroid receptor RNA activator protein (Srap), both coming from alternative splicing variants of the same gene [44]. These findings suggest that potentially some class 3 lncRNA could code for polypeptides, and some of the unknown transcripts that have evidence of protein coding potential could also have lncRNA functions. We saw potential of some transcripts predicted to be protein coding to have high sequence similarities with human or mouse lncRNA in this study. In lieu of this we ask, how many of our unknown transcripts predicted to have protein coding potential, can in fact be lncRNA that have been miss-annotated.

Examining conservation of lncRNA across species can assist in understanding how lncRNA have evolved and how natural selection pressures acting on lncRNA can determine function [36, 45]. The comparative analysis finds 289 (2.92%) and 119 (1.20%) class 3 putative lncRNA that had significant sequence similarities with human or mouse lncRNA respectively. Further, only a mere 36 (0.36%) class 3 putative lncRNA show significant sequence similarities with both a human and mouse lncRNA. This small number of lncRNA that are conserved with other species has also been seen in zebrafish, with 7 mouse and 9 human lncRNA mapping to only one of the 550 zebrafish lncRNA [46]. A recent study suggested that lncRNA sequences evolve rapidly and show very weak signatures of natural selection and are significantly less conserved than protein coding and non-coding regions of mRNA [36]. Our results support this.

A comparative analysis of lncRNA obtained from multiple bovine lncRNA studies show that many class 3 lncRNA overlap with annotated lncRNA across many studies that used RNA-Seq data, while in a paper that used EST we find very few class 3 lncRNA to overlap. This small overlap does not necessarily indicate that there is an error in the data or the annotations, rather it shows there is a need to have a standard method for determining lncRNA in bovine. Also lncRNA expression varies in different tissue types and the developmental stage of the organism, something we see in the validated blood lncRNA (Fig 7), and thus this could account for why not many lncRNA overlap.

There is a steadily increasing number of reported lncRNA that originate from pseudogenes. In human, 68 human pseudogenes were found to be transcribed and conserved in at least two other mammals [47]. One example of a pseudogene that codes for a lncRNA is the phosphatase and tensin homolog pseudogene 1 (*PTENP1)* that was discovered to code for an antisense RNA [48]. In the bovine genome only one study investigates the potential regulatory functions of pseudogenes, with the cytochrome P450, family 19, pseudogene 1 (*CYP19P1)*, is believed to code for a noncoding transcript that could interact with the protein coding gene cytochrome P450, family 19, subfamily A, polypeptide 1 (*CYP19A1)*, however an *in silico* study found no ncRNA coding sequences at that pseudogene [49]. Using a combination of expression patterns from the reconstructed RNA-Seq reads that aligned in and around pseudogene regions and by performing conservation analysis of the pseudogenes with high expression counts and known human and mouse lncRNA, we attempted to identify potential lncRNA candidates. It is important to note that although a pseudogene can show moderate or high expression and found abundantly expressed across many tissue samples, this can also be due to the mapping ambiguity of the RNA-Seq data to the pseudogenes. We found a total of 255 pseudogenes that have high expression counts along with significant sequence similarities with known human and mouse lncRNA. These represent the most likely to be lncRNA that arise from pseudogenes, however further studies will be required to determine potential functions and confirm their existence.

Predicting lncRNA transcripts located either upstream or downstream of genes is relatively difficult due to the added complexity that the potential lncRNA candidate could in fact be part of a protein coding gene. To get around this, we validated some of our class 3 lncRNA using stranded RNA-Seq libraries from liver (same cow as 18 tissue samples) and blood (3 additional cows). We find that many of the validated transcripts in both liver and blood show independent coding potential when compared to neighboring protein coding genes with a large portion having coding directions opposite to that of protein coding genes. In liver we see 43.48% of the validated class3 lncRNA found close to protein coding genes to have a opposite coding direction to the neighboring protein coding gene, while for the blood validated lncRNA we see 52.22% of the lncRNA found close to genes to have a opposite coding direction. These findings provide evidence that some of the class 3 lncRNA have the potential to be independently coded lncRNA, however further work is needed to find and isolate these transcripts that have opposite coding directions to protein coding genes for further lncRNA discrimination.

In addition, there is a trend for lncRNA to exhibit similar expression patterns to protein coding genes that they are located near to [7]. Based on this, correlation of expression patterns between the putative lncRNA with the nearby coding transcript would not provide evidence of lncRNA function, therefore more refined methods to determine lncRNA would be required. Moreover, polyA(+) captured RNA can contain degraded RNA products [50] and this can be seen as bias in transcript coverage towards the 3’ end of genes [51]. This constraint can impact our ability to distinguish RNA-Seq reads aligning near the 3’ end of a transcript as either potential lncRNA or just artifacts of this bias. Stranded RNA-Seq data is one potential solution that can help to validate if transcripts truly are independently coding transcripts. We believe that the liver and blood validated class 3 lncRNA in this study have the highest potential to be lncRNA and further analysis would need be undertaken to confirm their annotation.

On a final note, studies have used the underlying biological information of single nucleotide polymorphisms (SNP) to determine which groups of SNP are *a priori* enriched in dairy and beef complex traits [52]. With our bovine lncRNA catalogue, this type of analysis could be further extended to include these lncRNA classes to investigate if including this new class for variants found exclusively in lncRNA regions can potentially show enrichment for trait associated variants in complex bovine traits.

## Conclusion

We describe a catalogue of lncRNA using polyA(+) captured transcripts from RNA sequencing across 18 tissue samples. We found a total of 9,778 class 3 transcripts that showed no protein coding potential and had moderate to high expression in all three replicates in at least one tissue sample. A further 726 liver and 1,668 blood class 3 lncRNA were validated with stranded liver and blood libraries respectively. Long ncRNA are known to have diverse functions across many species, and studies are beginning to unravel some of the diverse functions lncRNA have and how they can ultimately impact complex phenotypes. Potentially a polymorphism in one of these lncRNA elements could have an effect on complex phenotypes and this is an area that still remains relatively novel in bovine genomics. However, more accurate and reliable annotations of both mRNA and lncRNA in the bovine genome will greatly assist in refining the location of such variants.

## Materials and Methods

### RNA extraction, tissue sampling, sequencing and alignment

Polyadenylated RNA from 18 tissues were collected from one lactating cow directly after euthanasia and sequenced. Blood tissues were also collected from an additional 3 lactating cows with stranded information. 40 to 100 million reads were generated per tissue sample in triplicate. On average 92% of reads aligned to the genome for each tissue, with more than 70% mapping uniquely across all tissues. BAM files have been submitted to NCBI Sequence Read Archive and can be found using study accession number [NCBI:SRP042639]. The tissues used in this study include; adrenal gland, black skin, white blood cells, caudal lobe of brain, brain cerebellum, heart, kidney, leg muscle (semimembranosus), liver, lung, intestinal lymph node, mammary gland, ovary, spleen, thymus, thyroid, tongue and white skin.

FastQC was used to assess sequence quality and in house scripts were used to filter poor quality bases and sequence reads. The, quality control, filtration, read alignment to the reference genome and generation of the SAM files for the 18 tissue samples were performed as described in another study [32].

### Ethics Statement

The cow from which 18 tissues samples were taken was euthanized for a reason other than collecting samples (humane grounds), the local Animal Ethics Committee (DEPI Agricultural Research and Extension Animal Ethics Committee) advised ethics approval was not required. For the other cows, blood samples were taken under approved ethics proposal 2012–13 (Animal Ethics Committee (DEPI Agricultural Research and Extension Animal Ethics Committee).

### Finding intergenic long noncoding RNA

Using SAM files from alignment described above we used a Cufflinks/Cuffmerge/Cuffcompare [23] pipeline to assemble transcripts for all three replicates in each tissue sample according to the Ensemble reference gene set release 75 [24] and compared them to the Ensembl gene GTF file (version 1.05). Cuffmerge was used to merge the transcript assemblies for all three replicates across all 18 tissues into one large final GTF file. We extracted entries in the final GTF file that had a class code of “u”, where u represents an unknown intergenic transcript, or “x”, where x represents an exonic overlap with the reference genome but on the opposite strand. Similar to [16] we used Cuffcompare to compare our transcripts to those in the NCBI iGenomes repository [25] as a further filtering step to remove transcripts that have partially supported protein sequences [16]. The UCSC utility twoBitTofa [53] was used to obtain the nucleotide sequences for the transcripts giving us a fastA file that was used for further comparative analysis.

### Long non-coding RNA filtering pipeline

To find transcripts most likely to be noncoding RNA transcripts, we created a pipeline to filter out the transcripts that had a high chance of being protein coding was developed. This was done in 3 steps, step one was an ORF analysis using EMBOSS getorf [54], step two was to use blastx (version 2.2.25+) that converted our nucleotide sequences to a protein sequence and compared the sequences to the non-redundant protein sequence database, and the third step was to use the tools CPC [26] and CNCI [27]. These steps will be outlined in detail in S1 Fig which shows the workflow of our filtering pipeline.

#### Stage 1. ORF Analysis

getorf from the EMBOSS software package [54] was used to find all possible ORF in both the forward and the reverse direction of the transcript. Each of the ORF was then treated as individual protein sequences for further analysis.

Next we determined possible protein coding domains for each of the ORFs from our unknown transcripts. A script was developed that ran blastp (version 2.2.25+) on all ORF in our dataset. This was to determine if any significant sequence similarity existed with the ORF sequence and a portion of the sequence in the nr database. An E-value of 1x10^-06^ was used as a cut-off. The script then grouped all ORF for each transcript together and determined if the transcript had any significant sequence similarities with any protein sequence in the database, if it didn’t then it was considered to be a potential lncRNA.

#### Stage 2. Blastx

We determined if our transcripts had any significant matches with protein sequences by using blastx (version 2.2.25+). Blastx is a tool from the blast package that converts a nucleotide sequence to a protein coding sequence and then blasts it against a protein sequence database to find a match. An E-value of 1x10^**-06**^ was used for the blastx analysis and we selected transcripts that did not show any significant matches with known protein coding sequences.

#### Stage 3. CPC and CNCI Tools

The third stage used the tools Coding Potential Calculator (CPC) version 0.9 [26] and CNCI [27] to predict the coding and noncoding potential of transcripts. The CPC tool is a support vector machine (SVM) and determines whether a given sequence has coding potential (a positive score) or noncoding potential (a negative score), the more negative the score the greater the probability the sequence is noncoding. A cut off score of < -0.5, similar to [16], was used to select for transcripts with noncoding potential for CPC while for CNCI we selected transcripts that had a negative “noncoding” prediction. Finally, we also aligned the transcripts to the protein domain database pfam [28] to determine any protein domains.

### Obtaining read counts, filtering of low read counts and differential expression analysis

The raw read counts for all tissues were obtained with the tool HTSeq [55], using a modified version of the cufflinks produced GTF (kept only the transcripts with unknown annotations) file as input and the SAM files for all replicates in all tissues. HTSeq was ran with default parameters, specifying for non-stranded (—stranded = no) and union mode (—mode = union) to get the counts matrix for each of the tissue samples. The results for each replicate were combined into a final single counts matrix for each tissue. Each of the tissue counts matrices were combined into a single large counts matrix file for all tissues and replicates. From this file we extracted only the class 3 putative lncRNA transcripts.

The final counts matrix file was used as input for the R package EdgeR [29] for normalization of the data, and to filter out transcripts that had very low read counts, only the transcripts that had at least a read count of 25 across all three replicates in a single tissue were retained.

A heat map of the Euclidean distance between tissues and replicates was generated using the packages DESeq [30] to normalize the counts expression matrix and determine co-expression of genes. For visualization of the data we used the standard R heatmap package.

Differential analysis was carried out by using DESeq in a tissue x tissue basis using the *nbinomTest* command by following the instructions from the DESeq reference manual. In house scripts were developed to automate the process of comparing each tissue and combined the output of *nbinomTest* into single csv files. From the differentially expression analysis files we determined upregulation for each tissue x tissue comparison if the log^2^ fold change was negative and the *P-value* is < = 0.05 (multiple testing corrected). Downregulation is determined if the log^2^ fold change was positive and the *P-value* was < = 0.05 (multiple testing corrected). This resulted in a matrix with upregulated transcript in the rows and the downregulated transcripts in the columns as shown in S2 Table. We used the R heatmap package to create the heatmap of this data.

To calculate the average number of differentially expressed transcripts, we calculated, the average upregulation and downregulation for each tissue from the differential expression matrix file (S2 Table), and plotted these values as a bar graph using excel.

### Homology analysis with ncRNA in human and mouse

Human and mouse ncRNA were obtained from; GENCODE v7 [33], NONCODE v4 [34] and lncRNAdb [35] databases. A bash shell script was developed that used blastn with an E-value of 1x10^-06^ to blast unknown transcripts with each of the human or mouse databases. From this we extracted the sequences that had significant hits or matches with a lncRNA present in the databases.

### Finding Pseudogene lncRNA

Similar to what was done in finding intergenic lncRNA, we used a Cufflinks/Cuffmerge pipeline to predict possible pseudogene lncRNA. Pseudogenes from the Ensembl v 1.74 gtf file were extracted using in house scripts and Cufflinks was used for transcript assembly with RNA-Seq reads that map to the pseudogene loci. Cuffmerge was used to combine all the output from Cufflinks into a final file that included all assembled transcripts across all replicates and tissues. EdgeR was used to filter out the pseudogene transcripts that had low read counts, keeping only the pseudogenes that had moderate to high expression levels.

We performed a comparative analysis to determine if any of our pseudogene transcripts had sequence similarities with known lncRNA from human and/or mouse lncRNA. Bash shell scripts were used to obtain the fastaA sequence for each pseudogene transcript (using tool twobittofa) and then performing a blastn search with an *E*-value of 1x10^-06^ each the lncRNA databases as described above, extracting all transcripts that had hits with a lncRNA from the databases.

## Supporting Information

S1 Fig Workflow of the filtering pipeline used in this study. (PDF)

S1 Table Sum of the number of fastq files generated per tissue and the number of RNA-Seq reads that paired uniquely to the reference genome. (XLSX)

S2 Table Total number of class 3 putative lncRNA. (XLSX)

S3 Table Matrix of upregulation and downregulation of class 3 putative lncRNA. (XLSX)

S4 Table Class 3 putative lncRNA with sequence similarities with human or mouse lncRNA. (XLSX)

S5 Table Class 3 putative lncRNA found to overlap or are near protein coding genes. (XLSX)

S6 Table Total number of blood validated class 3 lncRNA found, including those that overlap or are near protein coding genes. (XLSX)

S7 Table Total number of pseudogenes with potential lncRNA function. (XLSX)
