# Supplementary figures and images for: A catalogue of novel bovine long noncoding RNA across 18 tissues
Source: PLoS One. 2015 Oct 23;10(10):e0141225. doi: 10.1371/journal.pone.0141225 (PMC4619662; doi:10.1371/journal.pone.0141225)

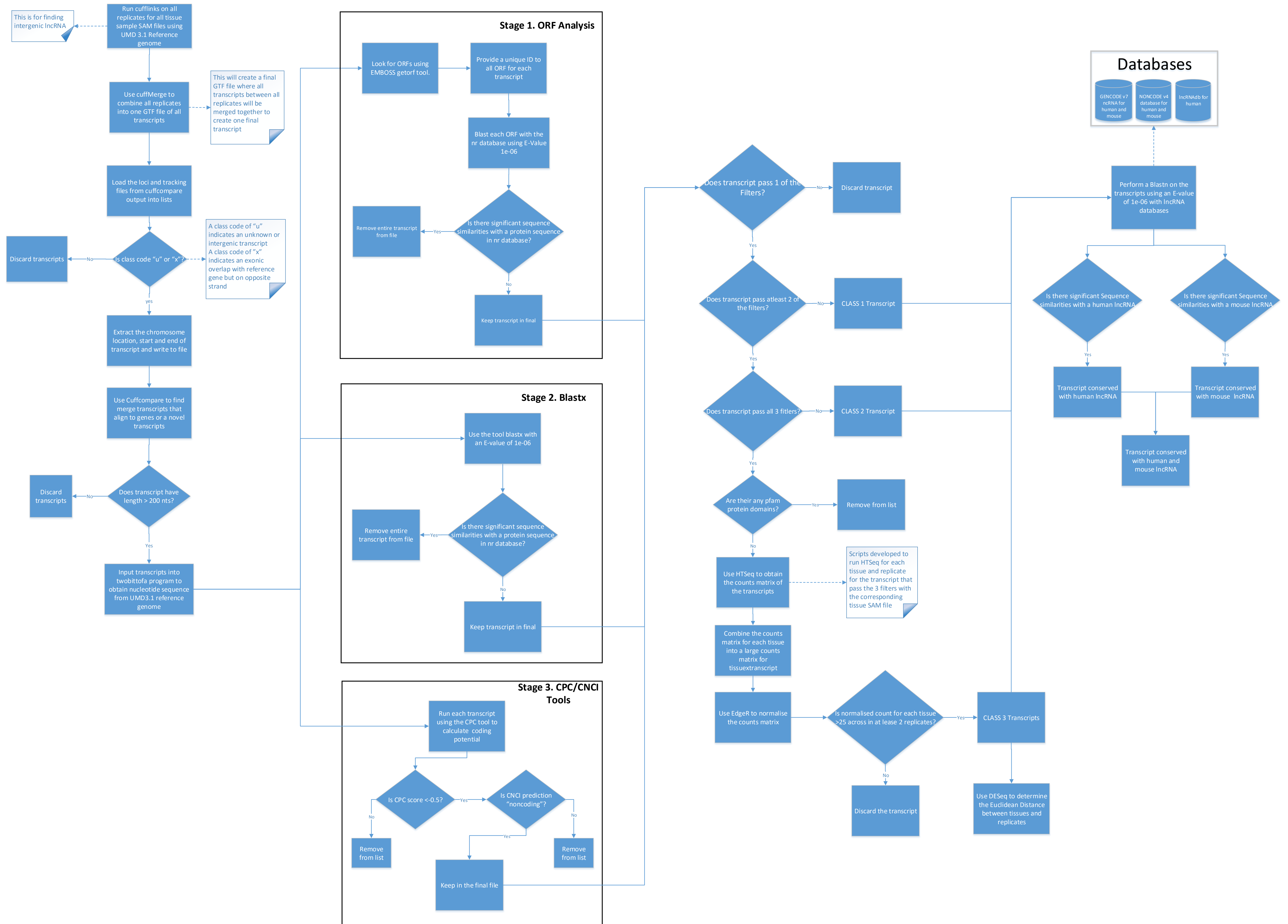

Supplement: S1 Fig — (PDF) [file pone.0141225.s001.pdf]
